# Evaluation of Global South’s efficiency at the Summer Olympics

**DOI:** 10.1371/journal.pone.0315054

**Published:** 2025-01-28

**Authors:** Julian Alexander Klöcker, Frank Daumann

**Affiliations:** Chair of Sports Economics and Health Economics, Friedrich Schiller University Jena, Jena, Germany; Universiti Malaya, MALAYSIA

## Abstract

States differ significantly in international sports competitions in how they use the resources they have and whether they do so in an efficient manner. In this paper, we investigate the efficiency of the nations from the so-called “Global South”, in total 52 states, during the 2000–2024 Summer Olympics. By doing this, our paper is the first using the Bayesian stochastic frontier analysis for exploring the performance of the states of the Global South. We perform an age decomposition, which shows that the 25–29 and 30–34 age cohorts contribute the most to Olympic performance. Our findings also suggests that transient efficiencies are higher than persistent efficiencies in a majority of the analyzed nations. Our analysis represents an important contribution in analyzing developing states‘ efficiency in elite sports.

## 1. Introduction

Nations are interested in the success of their athletes, no matter if it takes places in individual sports or in team sports, in international competitions. Research takes up this endeavor to some extent and deals with the determinants of sporting success at elite level from an empirical point of view [1]. It remains certain that national sports policies, just as companies operating on the market, must be committed to the economic principle: they need to act in an efficient way. In other words: a nation’s outcome at Olympic games may not be evaluated only based on the raw number of medals, but on the relation between outputs and inputs, too. The overwhelming quantity of previous efficiency analysis in sports have focused on either national leagues or across the nations at one particular multi-sport event. Our paper seeks to expand the literature and by doing this, our paper is the first that makes use of the Bayesian stochastic frontier analysis (BSFA) to evaluate the performance of the states of the so-called “Global South”. The term “Global South” refers to the developing and least developed countries located in Asia, Africa, Central/South America. These nations struggle with high income inequality, lower life expectancy and political instability [2]. In addition, these nations have considerable less economic potency in order to promote elite sports than the wealthier nations from Europe or North America.

Our motivation behind analyzing this particular set of countries is directly linked to the fact that these states gradually play an important role in the world. For instance, the economic drivers of the world economy have gradually shifted away from the advanced nations in 2000 (GDP growth rate at of the advanced economies at 4.1%, Africa 4.2%, South Asia 4.2%, Southeast Asia 5.8%, South America 3.2%) to the countries of the Global South in 2024 (GDP growth rate of the advanced economies at 1.5%, Africa 3.2%, South Asia 5.4%, Southeast Asia 4.2%, South America 1.6%) [3]. The question arises: What is the relation between the limited resources in the countries of the Global South to their Olympic performance? As far as we could identify, our study is the first that explicitly regards both, the performance and efficiency at the Summer Olympics of nations of the Global South, what emphasizes the relevance of our contribution; other studies accounting for developing nations do not focus on the entire Global South. For example, Hoffmann at al. [4] analyze the ASEAN members with focus on policy implications for Olympic games. A recent paper by Lin et al. [5] evaluates the efficiency of 85 nations participated in eight consecutive Olympic and Paralympic Games from 1992 to 2020. Among their set of countries, there are some nations from the Global South, too (21 out of 85) [5]. However, either such studies are not up to date or do not explicitly consider the countries of the Global South, such as, e.g., Rathke and Woitek [6]. Besides, all these papers consider the non-parametric data envelopment analysis [e.g., 5], which is lacking explanatory power by being non-stochastic, or use the standard stochastic frontier analysis [e.g., 6]. We in turn, adopt the BSFA that is relatively low frequented within the sports economics literature referring to efficiency measurements.

The reminder of the paper is structured as follows: We start with a theoretical background of international success at Olympics. Then we give a brief review of the existing literature on this topic, especially in sports economics. Section 4 provides the actual analysis. In the discussion, we will take the limitations into consideration. Finally, we close the paper with concluding remarks.

## 2. Theoretical background

Sport performance at international tournaments, especially the Olympics, have always been served as a tool of public relation for governments. To domestic observers, international success in sports foster the publicity of National Olympic Committees, the attractiveness of participating in sports and thus opens new consumer markets [7]. To foreign observers, success at the international stage increases the reputation of a nation and points toward the excellence of a political system. The determinants of sports performance can be partitioned into three levels, the micro, meso and macro levels [8]. While micro level determinants concern the close environment of athletes, meso level determinants are related to sports policy topics, e.g., corruption. In terms of sports policy and management, the allocation of capital within the sports system is suboptimal if administrations choose those projects that promise bribes and does not have the largest public benefit [9]. Administrations thus become unable or unwilling for efficient management tasks as a result of wasteful rent-seeking. Corruption, as a consequence of weak control instruments, harms the quality of fundings [9, 10]. Hence, corruption is supposed to negatively impact the sporting performance. The fruits from sports investments, e.g., the time in practice, are stolen by missing clear-cut defined and enforced property rights. Furthermore, [11] emphasize the value of structural conditions that adapt management in sports policy. The culture of corruption within a society has a negative impact on the performance at the Olympics, since corruption as well as nepotism lead to misallocations of financial resources and are essential for inefficient managements due to oversights and higher scrutiny [7, 12, 13]. Moreover, structural conditions affecting the sports policy management are supposed to be critical in nations suffering from corruption, nepotism and money leaks [14]. Factors such as the wealth and the population size of a nation are usually condensed at the macro level [5–7, 15, 16]. Economic potency is of superior meaning, because successful participations at Olympics require investments in sports. Wealthier nations are able to spend more public funds on training infrastructure, sports facilities or skilled coaching personal. Also an effective talent identification system is expensive and need to be financed in a long-term. Furthermore, it is well-known that welfare correlates with the general healthiness of a population due to better medical support. This is directly linked to the physical constitution of athletes. Conversely, the lower the income, the worse the medical and nutritional assistance [17]. The contribution of the population size is related to the fact that the larger the population, the larger the talent pool gets [18]. In addition, there is evidence that larger population size correlates with higher training quality; in terms of this, sports are often joint consumption goods and subsequently, talented athletes can strengthen their skills by training with highly talented partners [19]. Moreover, the importance of sports within a society is a crucial factor in explaining Olympic performance [6]. Importance might be approximated by the public funding of sports, because “flavor” for sports should go along with a society’s willingness for governmental expenditures on it.

## 3. Efficiency analysis and sports policy

Although the evaluation of efficiency is a common tool, recent studies distinguish between transient (or time-varying) and persistent (or time-invariant) inefficiency. While the first one is supposed to be mainly originated by non-systematic management or governance misdeterminations that may be fixed in a short-term, persistent inefficiencies are caused by structural problems and systematic malperformances by the management or government [20–22]. In the sports economic context, however, the differentiation between both forms of inefficiency is not established yet; only the non-distinguished term of “inefficiency” has been treated. Despite this, the detection of this dual feature of inefficiency is crucial to address appropriate policy implications.

While papers as [6, 23, 24] analyze the efficiency of a set of countries at several consecutive Olympics, the vast literature regarding the international level examine the efficiency of one particular National Olympic Committee at Olympic games. For example, Torres et al. [25] study the efficiency of the Spanish Olympic team at the 2008 Summer Olympics. Gulyas et al. [26] perform an efficiency analysis of the Hungarian Olympic National Committee at the 2016 Summer Olympics in context with governmental fundings. Several papers look on the differences across all participating nations at one specific Olympic contest. For instance, Lozano et al. [27] evaluate the nation’s performance at the 2021 Tokyo Games, Flegl and Andrade [28] analyze the 2016 Olympic games and Wu et al. [29] consider the 2008 Olympics. Though, a considerable share of efficiency studies focus on the club sports level. Frick and Simmons [30] evaluate the efficiency of management quality and its contribution on success in the German 1^st^ Bundesliga. Similar papers, e.g., [31, 32] for the Brazilian and Italian football leagues or [33] concerning the NHL, put the question of intra-league efficiency in the center of their research. In another way, Pyun et al. [34] analyze the efficiency of marketing strategies among the MLB teams during the last 20 years. This shows that an in-depth efficiency analysis of the countries of the global South in the Olympic Games is still pending.

## 4. Methodology

In contrast to the mass of earlier studies, we apply a Bayesian-based stochastic frontier estimation, because it provides some advantages over the traditional method. In the one hand, BSFA is resilient against estimation biases due to outliers. For example, Bayesian techniques enable us to estimate robust inferences even in cases a country’s medal count at one particular Olympic game is an outlier, as for example, in cases when nations had a home-advantage. In the other hand, the researcher may be constrained by small sample sizes that do not allow robust estimations. Since our sample is relatively small, only consisting of 364 observations (52 countries at 7 Olympics), the BSFA mitigates the problem of the small sample sizes:

"Bayesian methods appeal to researchers who only have access to a relatively small number of participants because Bayesian statistics are not based on large samples (i.e., the central limit theorem) and hence may produce reasonable results even with small to moderate sample sizes" [35, p. 3].

### 4.1 Variable selection

Our country set consists of ℐ≔{1,2,…,*N*} nations. We follow other authors such as [5–7, 27, 36] and use the GDP per capita and the population size as inputs. We retrieve data for both from the World Bank [37, 38]. While the previous research has always used the entire population size, we decompose it into four age classes, taking account for people aged 15–24, 25–29, 30–34 and 35–39. It allows us to assess the age-specific contribution on the performance, since the fraction of the population that usually attend the Olympics is within these ages.

In general, measuring the performance of the contestants as output variable is primarily considered in terms of medals. Though, such a strategy does not seem to hold for the performance evaluation of less successful nations or smaller countries appropriately, because larger populated nations have reasonably a higher probability to win a larger total of medals. Complementary, problems in measuring performances in multi-sports events occur by the variation of the probability to win among the particular types of sports due to the amount of competitions [17]. For example, there are more than 40 disciplines in athletics, and more than 30 in swimming, whereas in weightlifting, there are only 15 disciplines. To mitigate this source of biases, using the medal share instead of using absolute medal counts have recently been enforced (e.g., [6, 25, 27]). To bypass this issue, we construct an index based on the scope of the won medals, but weighed by the squad size [25, 39]. The squad size a nation is allowed to send does not need to be identical with the population size, since the delegation size is determined by many factors, such as qualification standards within a sports and quotas set by the IOC (The IOC allocates quotas for each sport to the National Olympic Committees based on efforts in contests in advance to the Olympics. This either limits or expands the number of athletes a country is allowed to send.). A large Olympic squad consequently reflects the competitiveness of a National Olympic Committee (NOC). Our index is then

$$\Lambda_{i}=\frac{\sum_{k}1/k}{Q_{i}}i\in I$$ (1)

with *k* = 1, 2, 3 we refer *Λ*_*i*_ as “Index of Effort”. The numerator describes the sum of all medal rankings, whereas the ranking gets inversely weighted (a gold medal gets one point, a silver medal gets ½ point, and so on). The denominator weighs the sum by the squad size *Q*_*i*_. As a result, we obtain an “Index of Effort per (athlete) capita” that is novel; [25, 39] use similar indices, but none of these can be interpreted as output per athlete. All medal results of the corresponding years are retrieved from the website “Olympic Analytics” [40]. In line with [7, 13], we control for corruption. The standard definition of corruption is the abuse of entrusted power in order to benefit privately therefrom. Since measuring corruption is bad by definition, we use the “Control for Corruption Index” provided by the World Bank, which depicts the perception public power is abused for private gain [41]. We add a covariate to control for the political stability within a nation. We assume that a stable political system is an important factor for athletes to stay and practice in a country. Reversely, unstable political environments force athletes to take care of themselves or their families. Effective training would then be impossible. Accompanying features of a stable political system is to guarantee the regulatory processes within a society, e.g., setting financial incentives for successful athletes or budget cuts for underperforming athletes, too [42]. For this purpose, we make use of the “Political Stability Index” provided by the World Bank [43]. Both measures deviations from a normalized perception. In order to logarithmize the Control of Corruption-Index and the Political stability-index, we normalize the normal perceptions on 100.

### 4.2 Model specification

In contrast to the standard stochastic frontier model, the Bayesian counterpart merges prior information—the researcher’s beliefs about the parameters of interest—and the observed information from the data. We are then able to calculate posterior distributions of the parameters [44]. Due to the marginal prior independence of the estimated effects, the Bayesian frontier distinguishes from a common stochastic frontier model. This independence permits an accurate estimate of a model consistent with economic regularity conditions [32]. The economic regularity condition, which ensures interpretable results of the deviations from the frontier as inefficiencies (interpretation as inefficiencies in cases of non-regular frontiers are very controversial [45]), strictly depends on the frontier’s functional form. For instance, a common Cobb-Douglas function ensures regularity of the production frontier [45]. Our empirical model is:

$$\log\;\Lambda_{it}=\underset{j}{\sum }\phi_{j}\;\log\;X_{ijt}+v_{it}+u_{it}\;\forall i\in I$$ (2)

with *j* = 1, …, 8 where *Λ*_*it*_ are the *Λ*-scores of each nation at *t*. The j input variables related to the i-th country at *t*, *X*_*ijt*_, are the Political stability-index, the Control of Corruption-index, the population sizes of the age classes 15–24, 25–29, 30–34 and 35–39, the GDP per capita and the one-time lagged GDP per capita. The reason why we control for the lagged per capita-income is based on the idea, that not only the wealth in the Olympic year determines the economic potency to finance infrastructure, staff and so on. In reality, planning, training and qualification take place in the preceding year, hence the economic potency in *t*−1 should be incorporated. As mentioned, Bayesian methods require to set prior distributional assumptions; following [46–48], our prior distributions are

$$\phi_{j}∼N({0,100}^{2}I)$$ (3.0)

$$u_{it}∼N^{+}(0,\sigma_{u}^{-2})$$ (3.1)

$$v_{it}∼N(0,\sigma_{v}^{-2})$$ (3.2)

$$\sigma_{u}^{-2}∼Gamma(5,10\;\log\;r)$$ (3.3)

$$\sigma_{v}^{-2}∼Gamma(0.5E-04,0.5E-04)$$ (3.4)

whereas *r* is a hyperparameter and reflects the researcher’s beliefs of the efficiency. In the present paper, we assume a weak informative prior of *r* and set it to 0.5 [47, 48]. Therewith, we ensure that all priors are proper and independent across all parameters [46, 48]. We run two distinct regressions, model I with transient inefficiencies and model II with persistent inefficiencies.

## 5. Results

The estimation results of the posterior means, the standard deviations and the 90% credibility intervals of the regression coefficients are displayed in Table 1. The models are run by applying Gibbs samplings with 50,000 iterations, whereas the first 10,000 are used as burn-in cycle.

**Table 1:** Bayesian estimates of (2), Summer Olympics, 2000–2024.

	Normal-half-normal with transient inefficiency (model I)			Normal-half normal with persistent inefficiency (model II)		
	Posterior mean	*σ*	90% CRI	Posterior mean	*σ*	90% CRI
Constant	-5.496	6.987	[-6.098; -4.893]	-8.893	6.653	[-9.466;-8.319]
Political Stability	0.529	1.277	[0.418; 0.639]	1.088	0.660	[1.031; 1.144]
Control Corrup.	0.672	1.627	[0.531; 0.812]	0.787	1.392	[0.667; 0.907]
Pop 15–24	0.030	0.052	[0.025; 0.034]	0.010	0.028	[0.007; 0.012]
Pop 25–29	-0.094	0.117	[-0.104; -0.083]	-0.003	0.050	[-0.007; 0.001]
Pop 30–34	0.096	0.115	[0.086; 0.105]	0.002	0.049	[-0.002; 0.006]
Pop 35–39	-0.023	0.050	[-0.027; -0.018]	0.020	0.028	[0.017; 0.022]
*GDP*_*t*_	-0.015	0.028	[-0.017; -0.012]	-0.006	0.012	[-0.007; -0.004]
*GDP*_*t*−1_	0.011	0.028	[0.008; 0.013]	0.008	0.011	[0.007; 0.008]
*σ*_*v*_	0.064	0.004		0.048	0.002	
*σ*_*u*_	0.209	0.009		0.426	0.040	
BIC	41.4			40.7		

Source: own calculations with data from [37, 38, 40–41, 43].

The elasticity of the Political stability-index is 0.529% under model I, while under model II, there is a larger effect at 1.088%. By means of that, the Index of Effort tends to rise by 0.529%, respectively 1.088%, by a one-percentage expansion of the Political stability index. The Control for Corruption-index differs between both models, too, with elasticities at 0.672% and 0.787%. As expected, covariates that act at the meso level do much more contribute to the persistent than to the transient efficiency. The age decomposition suggests marginal elasticities under both models, whereby the cohort of the 24–29 years old has negative impact (-0.094% and -0.003%). Considering the oldest cohort of the 35–39 years old, we only find a negative impact on the performance under model I (-0.023%), while under model II, there is a positive sign (0.02%). By summing up the cohort’s effect, we approximately find an overall positive effect of 0.009% under model I and an approximately total elasticity of 0.029% under model II. A rise in the GDP per capita by 1% goes along with a small decline in the Index of Effort by 0.015%, though, the lagged GDP per capita shows a positive sign (at 0.011%). Considering the model with persistent inefficiencies, the impact of GDP per capita in the year of the Olympics has a small negative effect, too, at -0.006%, whereas the lagged GDP per capita indicates an elasticity at 0.008%, supporting our assumption, that wealth increases the preparation and training in the pre-Olympic year. In general, even if small estimated coefficients seemed not to be very significant in translating into noticeable differences in a nation’s efficiency or Olympic performance, one has to keep in mind that dealing with elasticities gives information about relative changes and not absolute. Since we logged *Λ*, even minor differences may reflect considerable differences in the raw number of medals.

The standard deviations of the stochastic inefficiency components *σ*_*u*_ differ much between both model specifications: regarding model I, the inefficiency fluctuates with 0.209, in model II with 0.426 (we also run regressions with the median age included, but these regressions indicated much higher BICs at 47.3 and 46.1, respectively).

To draw on the estimates in Table 1, we provide the mean posterior transient and persistent efficiencies of each nation between 2000 and 2024.

Table 2 depicts the estimated posterior mean efficiencies under model I show few variations among the different countries, in contrast to the results under model II. While Jamaica has the highest mean transient efficiency at 93.6%, Kenya has the highest persistent efficiency at 97.1%. In general, we observe a much higher persistence than transient inefficiency that refers to constitutional and systematic governance problems.

**Table 2:** Posterior mean efficiencies and standard deviations, Summer Olympics, 2000–2024.

	Model 1		Model II	
	Posterior Mean	*σ*	Posterior Mean	*σ*
Afghanistan	0.886	0.051	0.871	0.030
Algeria	0.885	0.051	0.840	0.027
Argentina	0.882	0.051	0.825	0.027
Botswana	0.901	0.047	0.909	0.037
Brazil	0.877	0.052	0.794	0.036
Burkina Faso	0.879	0.052	0.855	0.027
Burundi	0.876	0.053	0.884	0.030
Cameroon	0.884	0.051	0.858	0.027
China	0.920	0.048	0.828	0.054
Colombia	0.887	0.052	0.838	0.028
Costa Rica	0.882	0.045	0.859	0.032
Cote d’Ivoire	0.905	0.045	0.900	0.028
Cuba	0.922	0.046	0.906	0.031
Dominican Republic	0.893	0.052	0.868	0.027
Ecuador	0.884	0.051	0.852	0.026
Egypt	0.871	0.054	0.811	0.031
Ethiopia	0.933	0.042	0.920	0.036
Gabon	0.889	0.054	0.892	0.038
Ghana	0.864	0.053	0.823	0.027
Guatemala	0.884	0.051	0.857	0.026
India	0.853	0.054	0.754	0.050
Indonesia	0.905	0.049	0.836	0.039
Iran	0.923	0.045	0.884	0.032
Jamaica	0.936	0.042	0.962	0.030
Jordan	0.892	0.050	0.875	0.028
Kazakhstan	0.901	0.050	0.868	0.027
Kenya	0.932	0.039	0.971	0.024
Kyrgyztan	0.902	0.049	0.906	0.030
Malaysia	0.880	0.051	0.828	0.028
Mauritius	0.884	0.053	0.885	0.040
Mexico	0.879	0.052	0.807	0.032
Mongolia	0.900	0.051	0.902	0.033
Morocco	0.880	0.051	0.833	0.027
Mozambique	0.878	0.049	0.860	0.027
Namibia	0.886	0.052	0.887	0.035
Niger	0.876	0.052	0.858	0.026
Nigeria	0.870	0.053	0.810	0.035
Paraguay	0.848	0.053	0.868	0.029
Phillippines	0.885	0.050	0.832	0.032
South Africa	0.876	0.053	0.818	0.030
Sri Lanka	0.870	0.053	0.837	0.026
Sudan	0.879	0.052	0.850	0.029
Syria	0.883	0.052	0.863	0.023
Tajikistan	0.903	0.049	0.904	0.029
Thailand	0.902	0.049	0.850	0.031
Togo	0.880	0.052	0.874	0.028
Tunisia	0.890	0.051	0.862	0.027
Turkmenistan	0.884	0.052	0.871	0.031
Uganda	0.888	0.050	0.861	0.028
Uzbekistan	0.905	0.049	0.873	0.028
Vietnam	0.875	0.052	0.811	0.033
Zimbabwe	0.895	0.048	0.895	0.028

Source: own calculations with data from [37, 38, 40–41, 43].

In the sake of better understanding, we plot the evolution paths of transient and persistent efficiencies for each country. In order to do regional comparisons, we split the entire country set into five world regions: North Africa, Southern Africa, Central/South America, Central/South Asia and Southeast Asia. In advance, we put emphasis on interesting overall aspects: The transient inefficiencies are more pronounced than persistent inefficiencies, except for Botswana (9.9% vs. 9.1%), Burundi (12.4% vs. 11.6%), Gabon (11.1% vs. 10.8%), Jamaica (6.4% vs. 3.8%), Kenya (6.8% vs. 2.9%), Kyrgyz Republic (9.8% vs. 9.4%), Mongolia (10% vs. 9.8%), or Paraguay (15.2% vs. 13.2%). The political instability and corruption have substantial impacts on efficiency, whereby Mexico and Ethiopia are deeper examined in the next part. Eventually, economic shocks have varying impacts across nations (e.g., Argentina, Uganda), which are also deeper explored in the regional context.

**Fig 1:**
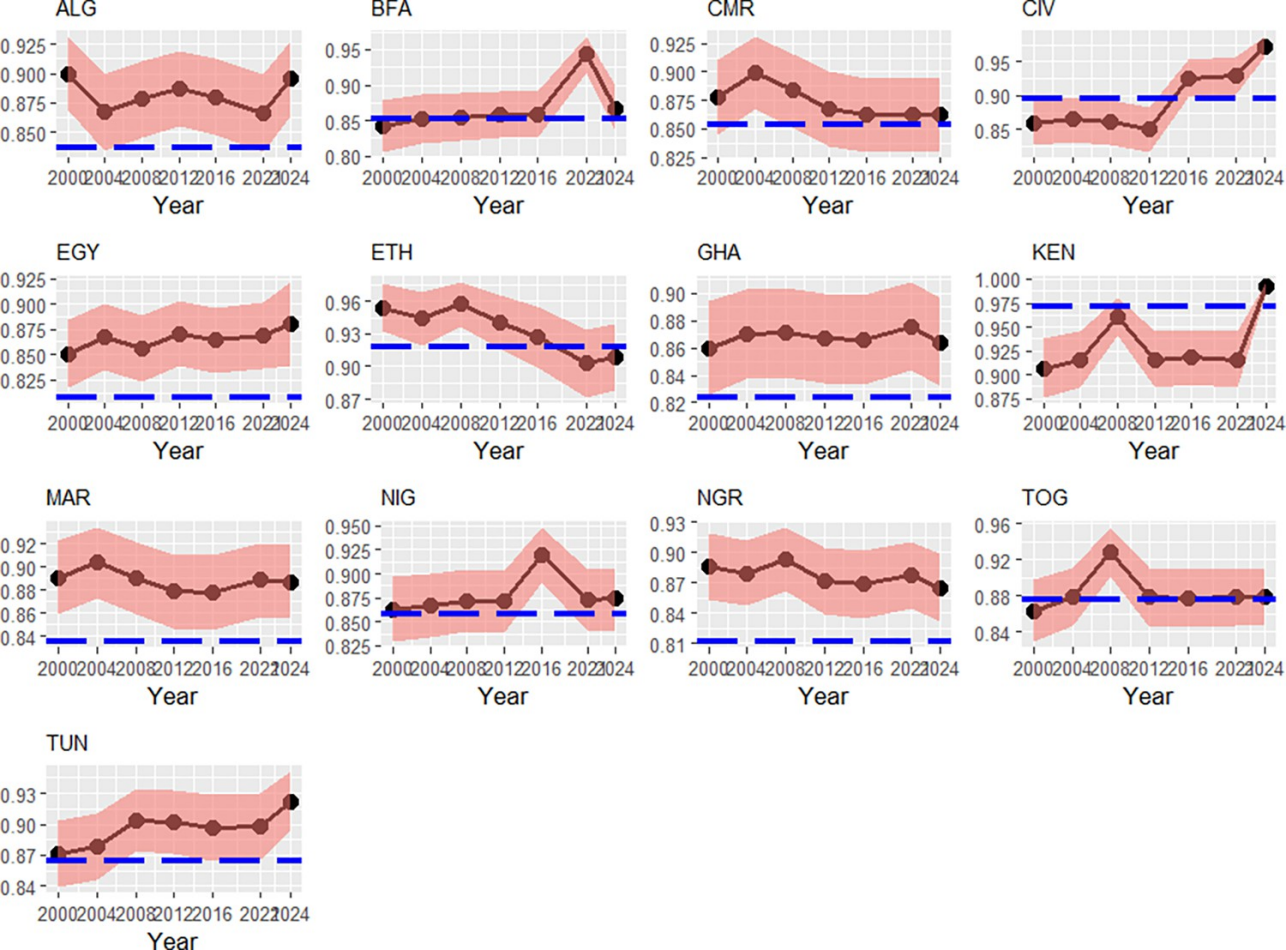
Transient efficiencies (black) and persistent efficiencies (blue), North Africa, 2000–2024. Note: Country codes are provided in the appendix. 90% credibility intervals. Source: own calculations with data from [37, 38, 40–41, 43].

Fig 1 shows the evolution of the transient efficiencies and the persistent efficiencies as well, for each country of North Africa. The paths of time-variant efficiencies have only decreased for Cameroon, Ethiopia, Marocco and Nigeria. Nations such as Egypt, Ivory Coast or Kenya have constantly improved their transient efficiency. An interesting fact is that only Kenya’s time-invariant efficiency is higher than the time-variant efficiency, suggesting that there is a well-performing sports policy with less systematic structural problems. Ethiopia’s transient efficiency has gradually declined. Reasons for this may be connected to bad decision-making, for instance bad training or disputes within the Ethiopian Olympic Committee. By looking on the BSFA results in Table 1, we can see that the Political stability-index affects the Index of Effort with an elasticity of 0.529%: indeed, Ethiopia has been in a long-lasting border conflict with its neighbor Eritrea. Tunisia’s rising transient efficiency may be associated with the demographic transition there: the four age-classes have grown at -0.38%, 0.22%, 0.55%, 0.8% on average between 2000 and 2024; hence, the contribution of the 3^rd^ age-class compensates the negative contribution of the 2^nd^ age-class.

**Fig 2:**
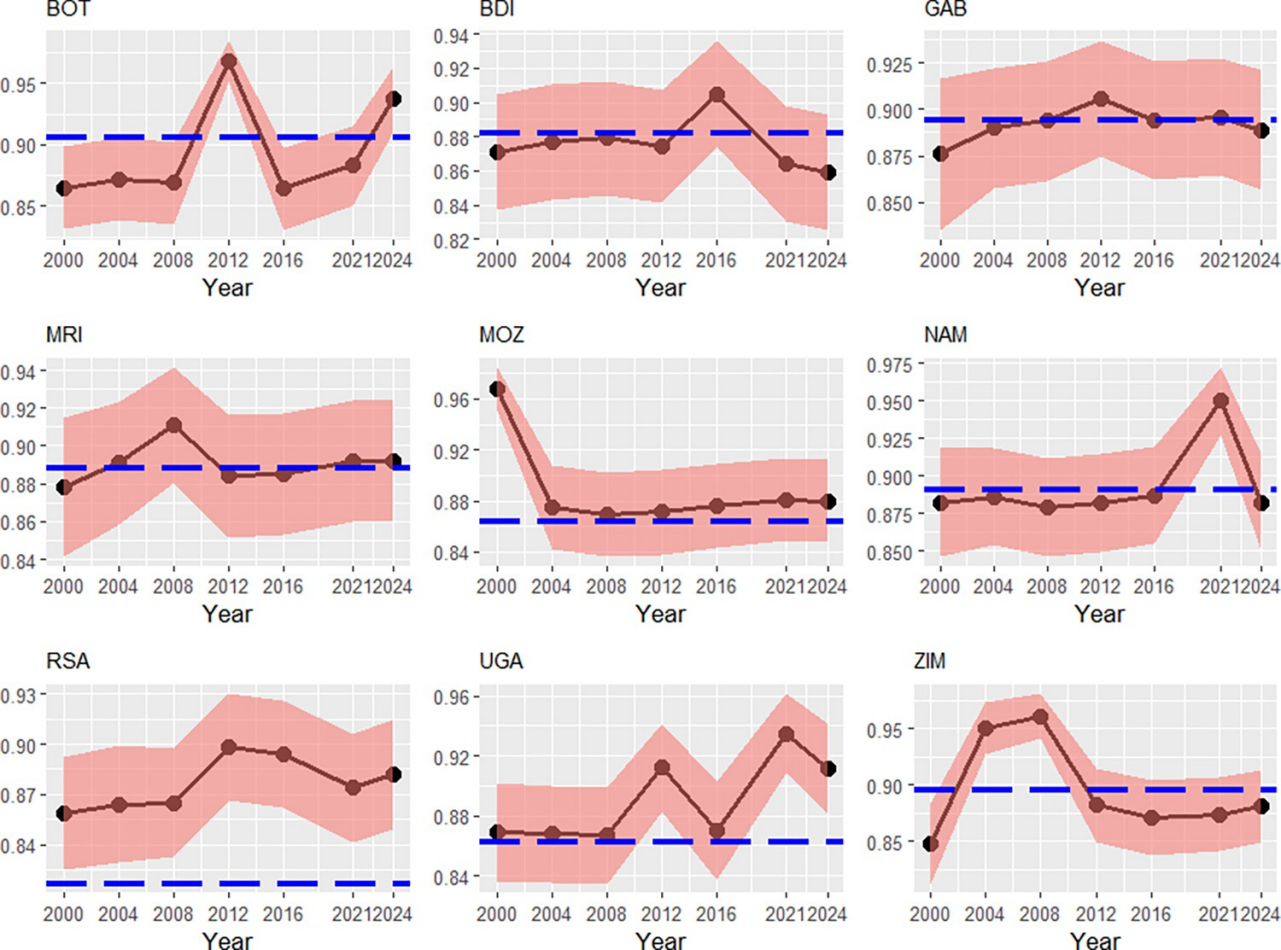
Transient efficiencies (red) and persistent efficiencies (blue), Southern Africa, 2000–2024. Note: Country codes are provided in the appendix. 90% credibility intervals. Source: own calculations with data from [37, 38, 40–41, 43].

In Fig 2, distinct to the North African nations, transient efficiencies have much more fluctuated across the countries. In the case of Mozambique, the 2000 floods with its severe implications shrunk the transient efficiency permanently. Uganda has faced off a more erratic path with upward trend. This upward trend might be induced by the two determinants GDP per capita (mean growth rate of 2.47% between 2000–2024 [37]), and the end of the Ugandan civil war in 2008, as well. The transient efficiency of Zimbabwe peaked in 2004 and 2008, although there were no outstanding economic or population growth at this time. Thus, we understand this as normal variation of the efficiency (*σ*_*u*_ = 0.209).

**Fig 3:**
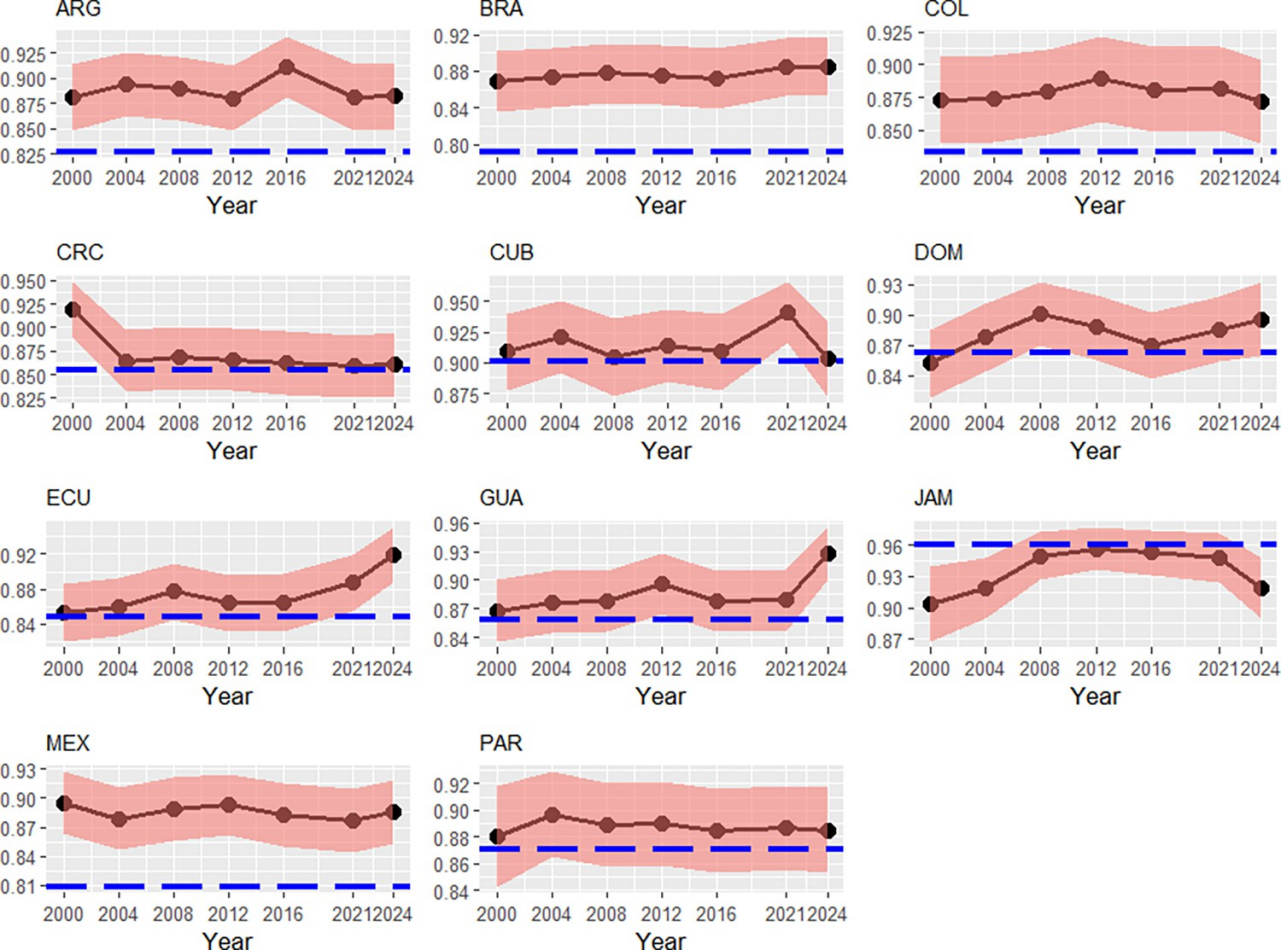
Transient efficiencies (black) and persistent efficiencies (blue), Central/South America, 2000–2024. Note: Country codes are provided in the appendix. 90% credibility intervals. Source: own calculations with data from [37, 38, 40–41, 43].

Fig 3 shows the evolution path of Jamaica’s time-varying efficiency illustrates a peak between the 2008–2016 Olympics, most probably based on the efforts in athletics. Argentina and Mexico have become less efficient since 2000, what in the Argentinian case should be seen in the light of the fragile economic situation with extreme inflation (this corresponds to a relative low persistent efficiency level). In the Mexican case, both, the Political stability-index as well as the Control for corruption-index have declined over the entire period. These results might be inherently associated with the drug war since the mid-2000s [41, 43], having led to a falling transient efficiency coupled with a low persistent efficiency level. Ecuador’s time-varying efficiency path has been upward trended since 2016 Olympics. Reasons may be a bettering political stability since this time (0.5 points).

**Fig 4:**
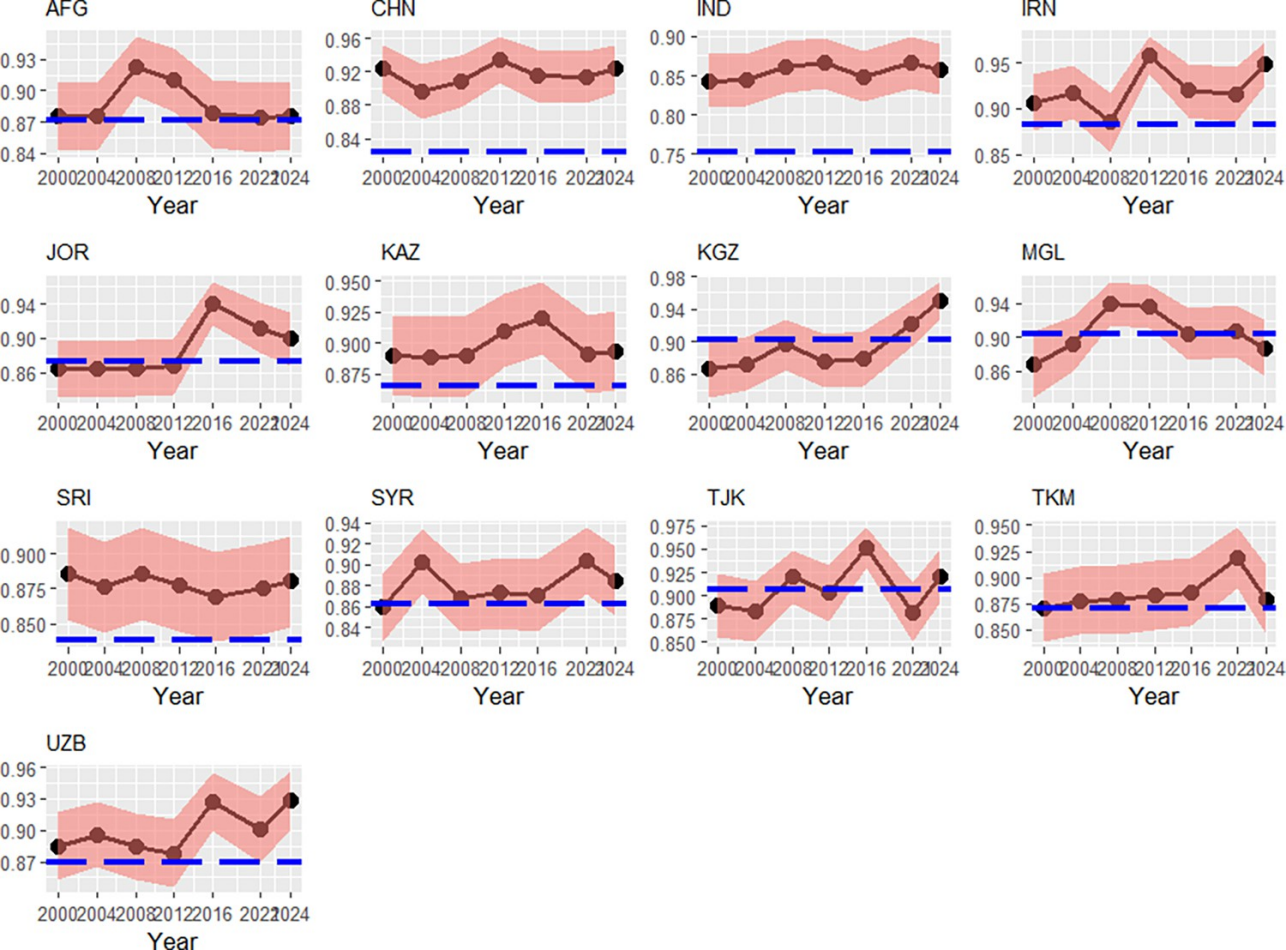
Transient efficiencies (red) and persistent efficiencies (blue), Central/South Asia, 2000–2024. Note: Country codes are provided in the appendix. 90% credibility intervals. Source: own calculations with data from [37, 38, 40–41, 43].

Looking at the efficiencies of countries from Central and South Asia in Fig 4, we see a lot of fluctuations across the countries. The former USSR-member Kyrgyzstan could steadily improve its time-varying efficiencies. An obvious explanation is the specialization in certain sports, e.g., Weightlifting [40]. Furthermore, e.g., in the Kyrgyz Republic, the 2^nd^ cohort has shrunk by 2.55% per year, but the 3^rd^ cohort has annually enlarged by about 0.21%. This two age classes hence affecting positively the Index of Effort and the time-varying efficiency. The GDP per capita has soared at 3.53% per year. We find a similar depiction for Uzbekistan, whereas the 2^nd^ cohort’s growth has been smaller than the 3^rd^ cohort’s (2% against 4.1%). Another factor that has led to an improved transient efficiency may be the upgraded political stability (+1.1 points). India has had an upward trending path, too. It has diversified its medal-winners-portfolio, especially with Shooting, Wrestling and Badminton [40], but it has also faced off a growth of the GDP per capita at about 3.1% annually [37] and an overall growth of the relevant age-classes at about 1.3%. This has permitted a larger faction of the population to practice sports and has improved the health system in India. Remarkably, we find the lowest persistent efficiency for India (75.4%). The question arises whether the society structure of the caste system tend to exclude a large fraction of potential athletes from doing sports, what consequently had to lead to relatively high time-invariant inefficiency. As Thorat and Newman [49] or Thorat and Madheswaran [50] show, the caste system indeed gives rise to decreasing efficiency in the job and perpetuate inequality.

**Fig 5:**
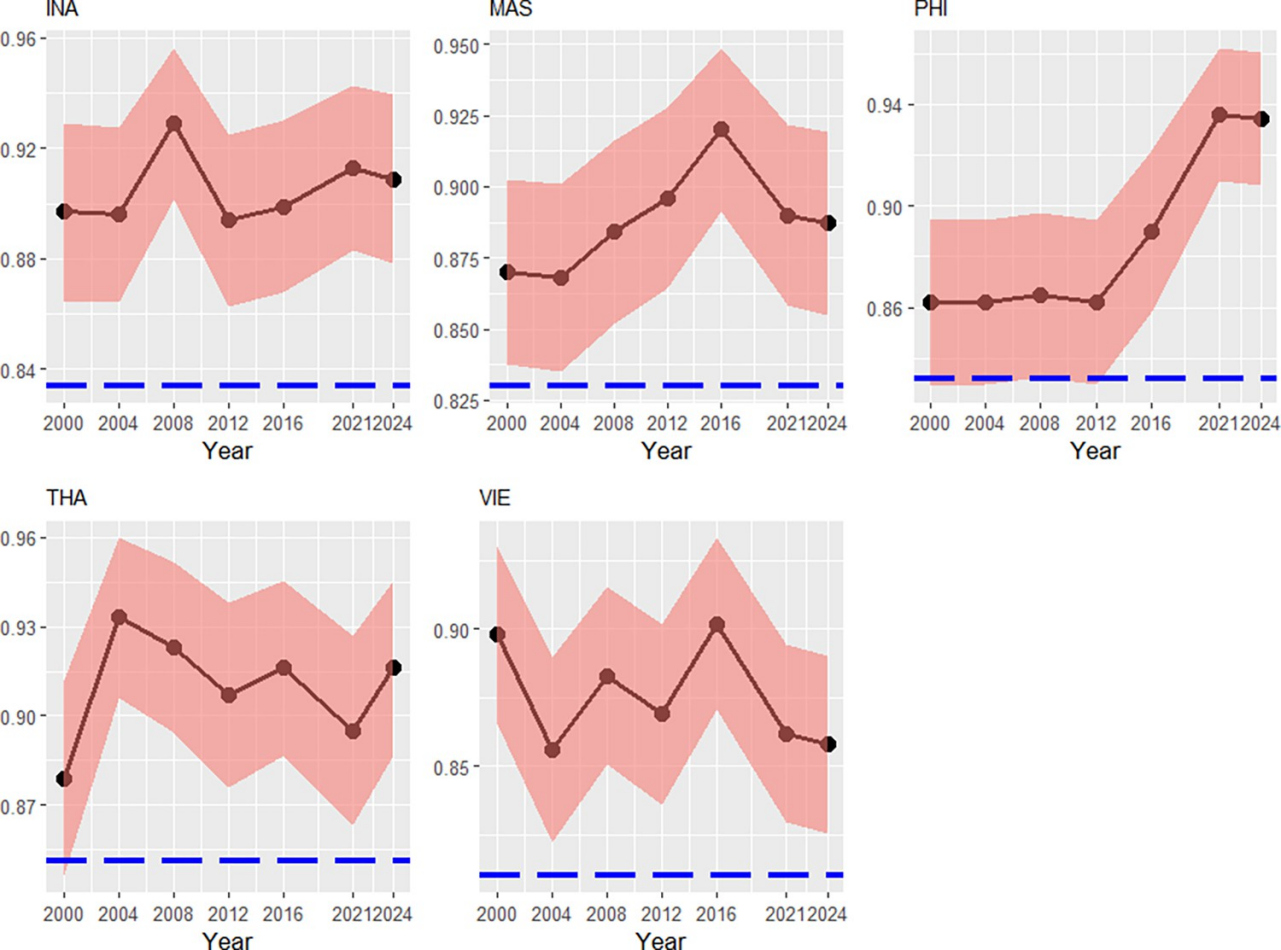
Transient efficiencies (red) and persistent efficiencies (blue), Southeast Asia, 2000–2024. Note: Country codes are provided in the appendix. 90% credibility intervals. Source: own calculations with data from [37, 38, 40–41, 43].

According to Fig 5, the evolution path of Philippines’ transient efficiency has trended upwards since the 2012 Olympics: the reasons might be, in the one hand, by specializing in two sports with several tournaments (boxing and weightlifting [40]), and in the other hand by a climbing political stability (until 2012–1.7 points, then it has risen up by 0.8 points). Thailand’s transient efficiency peaked in 2004, after then it has continued to decline. We can detect several sources: the contribution of each age-class (the 1^st^ cohort has grown at -0.31%, the 2^nd^ cohort at 0.9% and the 3^rd^ at 0.7%), where the contribution of the 3^rd^ age-class has been outweighed by the negative contribution of the 2^nd^ one. In addition, the political stability suffered during 2008–2016 and fell within this interval by 0.6 points). Among the Southeast Asian countries, Vietnam has indicated the lowest persistent efficiency. Causes might be a pronounced corruption level, though, this should be only one side of the coin: the Control for Corruption-index is not exceptionally high compared to the other Southeast Asian nations. Instead, since Vietnam still has a socialist regime, the socialist-like decrepit structures could be key factors of the high time-invariant inefficiency [51].

In Table 3, we provide an overview of results for each region.

**Table 3:** Overview of results for each region by average transient efficiency (Model I), average persistent efficiency (Model II) and key insights.

Region	Model I	Model II	Key Insights
North Africa	88.84%	86.27%	• Kenya’s time-invariant efficiency is higher than the time-variant efficiency, suggesting that there is a well-performing sports policy with less systematic structural problems. • Ethiopia’s transient efficiency has gradually declined. ○ Reasons for this may be connected to bad decision-making, for instance bad training or disputes within the Ethiopian Olympic Committee. ○ Ethiopia has been in a long-lasting border conflict with its neighbor Eritrea. • Tunisia’s rising transient efficiency may be associated with the demographic transition. ○ The four age-classes have grown at -0.38%, 0.22%, 0.55%, 0.8% on average between 2000 and 2024; hence, the contribution of the 3rd age-class compensates the negative contribution of the 2nd age-class.
Southern Africa	88.58%	87.67%	• Transient efficiencies have much fluctuated across the countries. • The 2000 floods in Mozambique with its severe implications shrunk its transient efficiency permanently. • Uganda has showed an erratic path with upward trend. ○ This might be induced by the GDP per capita (mean growth rate of 2.47% between 2000–2024). ○ The end of the Ugandan civil war in 2008 might improve the efficiency, too. • The transient efficiency of Zimbabwe peaked in 2004 and 2008, although there were no outstanding economic or population growth at this time. Thus, we understand this as a normal variation of the efficiency (*σ*_*u*_ = 0.209).
Central/South America	88.85%	86.19%	• The evolution path of Jamaica’s time-varying efficiency peaked between the 2008–2016 Olympics, most probably based on the efforts in athletics. • Argentina has become less efficient since 2000, which should be seen in the light of the fragile economic situation with extreme inflation (this corresponds to a relatively low persistent efficiency level). • In the Mexican case, both, the Political stability-index as well as the Control for corruption-index have declined over the entire period. ○ These developments might be inherently associated with the drug war since the mid-2000s, having led to a falling transient efficiency coupled with a low persistent efficiency level. • Ecuador’s time-varying efficiency path has been upward trended since the 2016 Olympics. Reasons may be an imporved political stability since this time (0.5 points).
Central/South Asia	89.40%	86.85%	• Former USSR-member Kyrgyzstan could steadily improve its time-varying efficiencies. ○ One explanation is the specialization in certain sports, e.g., Weightlifting. ○ Furthermore, the 2nd cohort has shrunk by 2.55% per year, but the 3rd cohort has annually enlarged by about 0.21%. Hence, these two age classes affect positively the Index of Effort and the time-varying efficiency. ○ The GDP per capita soared at 3.53% per year. • There is a similar picture for Uzbekistan. ○ The 2nd cohort’s growth has been smaller than the 3rd cohort’s (2% against 4.1%). ○ Another factor that has led to an improved transient efficiency may be the upgraded political stability (+1.1 points). • India has had an upward trending path. ○ It has diversified its medal-winners-portfolio, especially with Shooting, Wrestling and Badminton. ○ It faced a growth of the GDP per capita at about 3.1% annually and an overall growth of the relevant age-classes at about 1.3%. ○ These developments have permitted a larger faction of the population to practice sports and has improved the health system in India. ○ We find the lowest persistent efficiency for India (75.4%). The question arises whether the society structure of the caste system tends to exclude a large fraction of potential athletes from doing sports, what consequently had to lead to relatively high time-invariant inefficiency.
Southeast Asia	88.94%	84.14%	• Philippines’ transient efficiency has trended upwardly since the 2012 Olympics. ○ One reason might be by specializing in two sports with several tournaments (boxing and weightlifting). ○ The climbing political stability (until 2012–1.7 points, then it has risen up by 0.8 points) might have improved the efficiency. • Thailand’s transient efficiency peaked in 2004, after then it has continued to decline. ○ The contribution of each age-class (the 1st cohort has grown at -0.31%, the 2nd cohort at 0.9% and the 3rd at 0.7%), where the contribution of the 3rd age-class has been outweighed by the negative contribution of the 2nd one. ○ The political stability suffered during 2008–2016 and fell within this interval by 0.6 points. • Vietnam has indicated the lowest persistent efficiency. ○ One cause might be a pronounced corruption level, however, the Control for Corruption-index is not exceptionally high compared to the other Southeast Asian nations. ○ Since Vietnam still has a socialist regime, the socialist-like decrepit structures could be key factors of the high time-invariant inefficiency.

## 6. Discussion

Our findings show that controlling for corruption as well as political stability considerably increase the Index of Effort under both models. These results are instructive: both, political stability and corruption are, at the first glance, structural occurrences. Both deteriorate the efficiency within administration and governance structures [28]. The political system can be affected by shocks (coups, government crisis), leading to fundamental breaks within the constitution of the political system. However, such shocks are singular events of disorder and the political system re-stabilizes after certain time, either due to political concessions or by much more restrictive policy. Our outcomes give rise to some policy implications for those nations suffering from higher persistent inefficiency in terms of long-term structural changes within their sports administrations. For instance, the culture of corruption is continual and may not be changed in short term; government’s actions to push back corruption and keeping the political system as stable as possible should improve the Olympic performance. Besides, countries with higher transient inefficiency might need targeted short-run governance reforms within the sports administrations, e.g., in cutting off red tapes.

We carry out an age decomposition to the population size. Contrary to the majority of other efficiency studies, which found evidence for a positive impact of the population size, our age-specific estimations indicate some ambivalence. The contributions of the age classes 15–24 and 35–39 are small in relation to the cohorts 25–29 and 30–34, whereby the cohort 25–29 affects the performance negatively. We detect some explanations: i) in some sports, e.g., gymnastics, the athletes are relatively young and peaked their induvial performance when they are at the end of the age-class 15–24. If countries do have a large pool of gymnasts in the age-group 25–29, but these have already crossed their performance peak, this cohort would negatively affect the performance in gymnastics. ii) usually, the number of athletes a NOC is allowed to send to any sports is restricted by quotas. In cases where the age distribution (of certain sports) has its density mass in the cohort 24–29 years old and additionally, a significant fraction of athletes achieves the qualification norm, the bulk of athletes sent to the Olympics would come from this cohort (that averagely crossed its performance peak). However, the overall demographic effect is, analog to former papers [e.g., 6–9, 23–27], positive.

Several of our estimations are consistent with results from older studies. For example, our relative high efficiency estimates for Cuba and Jamaica could also be found for the Summer Olympics between 1984 and 2000 by [24]. Albeit our results are distinct for the impact of the GDP per capita: we find a small negative elasticity in contrast to many former studies [e.g., 6, 15, 16, 29]. We account for the lagged GDP per capita, in order to check our assumption, that the economic situation in the year preceding the Olympics is important, too. The results verify this hypothesis. Regarding the efficiencies, only few nations have higher persistent than transient efficiencies, which induce systematic and structural problems in the sport governance.

Though, making clear statements about the differences in the evolution of the efficiencies are quite idle, because numerous institutional changes could have taken place and caused structural breaks that have made the Olympic performance more efficient. Also the importance of sports may vary across cultures and societies and consequently would affect performance and efficiency as well.

## 7. Limitations

To the truth belong some limitations. We abstain from including a covariate for sport fundings in our empirical model, because there is no data about sport fundings and secondly, we make use of the common proxy (the delegation size) in the denominator of our dependent variable Λ_*it*_. If we would have included the squad size as covariate, too, statistical concerns such as interdependency could have been arisen. However, the squad size as proxy for sport fundings is usual in the sport efficiency evaluation and plays the role of transmitting the compound impact of a country’s economic potency that finds expression in the volume of investments in sports [52]. We ignore any index that captures the importance of sports, unless we are aware of the consequences (e.g., omitted variables bias). The reason is straightforward, because there is no balanced panel data about the importance of sports within the societies of our country set. Utilizing the public funding of sports as proxy for the importance could not be operationalized caused by lacking data. A further aspect to mention is the choice of the weak informative prior distribution of the hyperparameter *r*. We have dispensed with a more detailed explanation of the different results when using various priors, as the model with the lowest BIC is to be selected with *r* = 0.5. Implementing a noninformative prior would not make sense, because our belief about the mean efficiency were then <0.5, implying a too low efficiency level across the countries.

## 8. Conclusion

The aim of our paper was to measure how the nations of the so-called “Global South” have been performed at the Summer Olympics since the 2000 Olympics. For that reason, we analyzed both, the transient and persistent efficiencies of these countries by a Bayesian stochastic frontier analysis. Reflecting that there has been a lack of research dealing exclusively with nations of the “Global South” and considering that former studies, such as [6], set focus on Olympic games in the 20^th^ century, we filled this research gap and thus essentially contributed to the body of literature. Furthermore, our paper distinguishes from earlier studies in the way that we provide evolution paths of time-varying efficiencies and control for different age-classes, as well. Additionally, our results have some selected political implications. Question of development economics are addressed in the sense of providing not only monetary transfers, but rather transfers in management and sports governance knowledge. Since mismanagement is supposed to be an essential source of persistent inefficiency in several countries, such knowledge transfers could indeed enhance the performance of managing authorities. Furthermore, knowledge interchangeability due to scientific cooperations and networks may be an alternative concept, from which all partners could benefit. Since many developing nations lack good sports infrastructure, such as modern equipment or ramshackle sports halls, Official Development Assistance by NOCs may progress the training opportunities in these countries. Eventually, as sports have ever been seen as connector of cultures, international relations among the developed countries and the Global South would improve equal sports partnerships and participation. This kind of equal partnership may be one main component to bring a culture of incorruption into the corruption-prone governance structures in these states. We encourage further research in the Olympic performance analysis of nations of the Global South by including covariates to control for the anchoring of sports culture or the quality of training infrastructure within the countries. Another interesting step forward would lay in a comparison of the Olympic efficiency between both blocks, the Global South and North.

## Supporting information

S1 File Summary statistics of covariates. (DOCX)

S1 Data (XLSX)
