# Supplementary material for: Evaluation of Global South’s efficiency at the Summer Olympics
Source: PLoS One. 2025 Jan 28;20(1):e0315054. doi: 10.1371/journal.pone.0315054 (PMC11774382; doi:10.1371/journal.pone.0315054)
Supplement: S1 File — (DOCX) [file pone.0315054.s001.docx]

**Summary Statistics of Covariates**

Pop 15-24

Min Mean Median Max sd

183299 15582335 4433585 258395278 41913429

Pop 25-29

Min Mean Median Max sd

92373 7220475 1755353 12224465 19951734

Pop 30-34

Min Mean Median Max sd

85180 6926232 1531391 125070136 19957069

Pop 35-39

Min Mean Median Max sd

69283 6381862 1302151 126364053 19109098

GDP per Capita

Min Mean Median Max sd

122 3711 2728 16595 3336

Index of Political Stability

Min Mean Median Max sd

97.1 99.4 99 101.2 1

Index of Corruption

Min Mean Median Max sd

98.2 99.4 99 101 1
